# Ellenbogengelenkluxation: Häufige Begleitverletzungen und aktuelle Therapiekonzepte

**DOI:** 10.1007/s00113-023-01318-9

**Published:** 2023-04-20

**Authors:** Lisa Klute, Leopold Henssler, Volker Alt, Maximilian Kerschbaum

**Affiliations:** Klinik und Poliklinik für Unfallchirurgie, Universitätsklinikum Regensburg (UKR), Franz-Josef-Strauss-Allee 11, 93053 Regensburg, Deutschland

**Keywords:** Gelenkinstabilität, Kollateralbänder, Ruptur, Knochenfrakturen, Reposition, Joint instability, Collateral ligaments, Rupture, Fractures, bone, Reduction

## Abstract

Luxationen des Ellenbogengelenks sind nach Schultergelenkluxationen eine der häufigsten Luxationsverletzungen am menschlichen Körper und stellen wegen ihrer Begleitverletzungen und Komplikationen weiterhin eine Herausforderung im klinischen Alltag dar. Betroffen sind v. a. junge Erwachsene, die sich während ihrer sportlichen oder alltäglichen Tätigkeiten verletzen. Unterschieden wird i. Allg. zwischen einer einfachen Ellenbogenluxation und einer Ellenbogenluxationsfraktur. Eine einheitliche Klassifikation oder ein Therapiealgorithmus hat sich jedoch insbesondere für die einfache Ellenbogenluxation mit den damit verbundenen ligamentären, muskulären und kapsulären Begleitverletzungen noch nicht durchgesetzt. Aufgrund dessen und wegen der Komplexität dieser Verletzung bedarf es eines standardisierten Vorgehens, um frühzeitig die optimale Therapie zu initiieren und den schmalen Behandlungspfad zwischen drohender chronischer Instabilität und Ellenbogensteife richtig auszuwählen.

## Abstract (en)

Dislocations of the elbow joint are among the most prevalent dislocation injuries in the human body after shoulder joint dislocations and represent a challenge in the clinical routine because of the concomitant injuries and complications. They predominantly affect young adults who become injured during athletic or daily activities. A distinction is generally made between a simple elbow dislocation and a dislocation fracture of the elbow; however, a uniform classification or treatment algorithm has not yet been established, especially for simple elbow dislocations with associated ligamentous, muscular and capsular concomitant injuries. Due to this and the complexity of this injury, a standardized approach is needed to initiate the optimal treatment at an early stage and to correctly select the narrow treatment pathway between impending chronic instability and elbow stiffness.

## Lernziele

Nach der Lektüre dieses Beitrags

- können Sie die wichtigen anatomischen Strukturen am Ellenbogengelenk benennen.
- können Sie Akutversorgung beim luxierten Ellenbogengelenk sicher durchführen.
- können Sie eine Verletzung am Ellenbogengelenk richtig beurteilen.
- schätzen Sie adäquat das Risiko von möglichen Komplikationen als Folge der Verletzung ein, um eine optimierte Therapie durchführen zu können.

## Einleitung

Die jährliche Inzidenz der Ellenbogenluxation in Europa beträgt etwa 6/100000 [1]. Betroffen sind Jugendliche und junge Erwachsene in einem Durchschnittsalter von etwa 30 Jahren [1]. Am häufigsten treten posteriore Luxationen nach einem Sturz auf den betroffenen Arm auf. Bei richtiger Therapieindikation sind die Behandlungsergebnisse der **einfachen Ellenbogengelenkluxation**

einfachen Ellenbogengelenkluxation

als sehr gut bis ausgezeichnet einzuschätzen [2]. Die Berichte über Begleitverletzungen zeigen, dass ungefähr 50 % der Ellenbogenluxationen mit Frakturen assoziiert sind [3]. Das zu erwartende Therapieergebnis der **Luxationsfrakturen**

Luxationsfrakturen

ist schlechter als das der einfachen Luxationen [4]. In den meisten Fällen kann eine einfache Luxation nach durchgeführter Reposition, kurzzeitiger Ruhigstellung und frühfunktioneller Beübung ausreichend therapiert sein, jedoch birgt insbesondere diese Verletzungsentität die Gefahr von übersehenen Rupturen der kapsuloligamentären Strukturen, die in **chronischen Instabilitäten**

chronischen Instabilitäten

resultieren können [5, 6].

## Anatomie und Stabilitätsmodelle

Die Kenntnis des detaillierten anatomischen Aufbaus des Ellenbogengelenks, der Kapsel-Band-Strukturen und der umgebenden Muskeln sollte als Grundlage der Behandlung von Verletzungen dienen. Medial bildet das **Humeroulnargelenk**

Humeroulnargelenk

den Eckpfeiler der knöchernen Stabilität und Mobilität in der Flexions- und Extensionsebene. Das **Olekranon**

Olekranon

bildet den Trizepsansatz im proximalen Bereich und geht im distalen Verlauf in die Diaphyse der **Ulna**

Ulna

über. Der **Processus coronoideus**

Processus coronoideus

(Koronoid) spielt mit seinen 2 diskreten Gelenkfacetten anterior eine essenzielle Rolle für die Stabilität des Gelenks. Das **Tuberculum subliminus**

**Figure 1:**
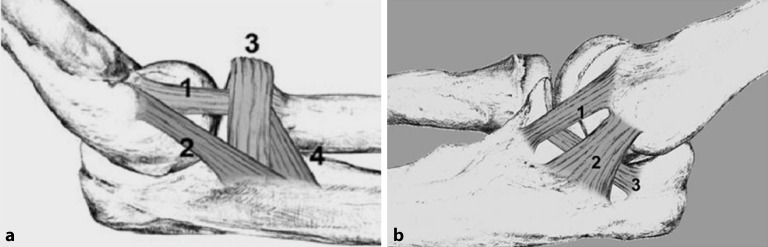

Tuberculum subliminus

ist ein wichtiges Element des medialen Anteils des Koronoids, an dem das starke anteriore Bündel des medialen ulnaren Kollateralbands (MUCL) ansetzt. Die Abb. 1 zeigt das MUCL mit dem anterioren, posterioren und transversen Bündel [7]. Lateral besteht das Ellenbogengelenk aus Radiuskopf und Capitulum und wird durch das **laterale Kollateralband**

laterale Kollateralband

(LCL) gegen Varusstress geschützt [6]. Als Teil des LCL verläuft das laterale ulnare Kollateralband (LUCL) vom lateralen Epicondylus bis zur Crista supinatoria der Ulna und trägt den Radiuskopf wie in einer Hängematte. Die Fasern des lateralen radialen Ligaments (RCL) ziehen vom Epicondylus lateralis humeri zum Lig. anulare, das den Radiuskopf annähernd zirkulär umgibt. Vom Lig. anulare zieht ein Teil des Bands als Lig. collaterale laterale accessorius zur Ulna (Abb. 1; [7]).

Gegen **Varusstress**

Varusstress

stabilisiert das Humeroulnargelenk den Ellenbogen zu 55 % in Streckung und 75 % in 90°-Beugung [8]. Der **Radiuskopf**

Radiuskopf

kann je nach Läsion des medialen Bandapparats, das hauptsächlich gegen Valgusstress stabilisiert, bis zu 30–75 % dieser Funktion übernehmen [8]. Außerdem überträgt der Radiuskopf bis zu 60 % der axialen Stabilität [6]. Kräfte, die nach posterior gerichtet sind, werden vom Processus coronoideus ausgeglichen.

Zu den **primären Stabilisatoren**

primären Stabilisatoren

nach O’Driscoll gehören das Humeroulnargelenk sowie der mediale und laterale Bandapparat [9]. Zu den **sekundären Stabilisatoren**

sekundären Stabilisatoren,

die nach einem Versagen der primären Stabilisatoren eine wichtige Rolle erlangen, zählen der Radiuskopf, die anteriore Kapsel sowie die Flexoren- und Extensorenmuskulatur, die das Ellenbogengelenk umgibt [9].

**Figure 2:**
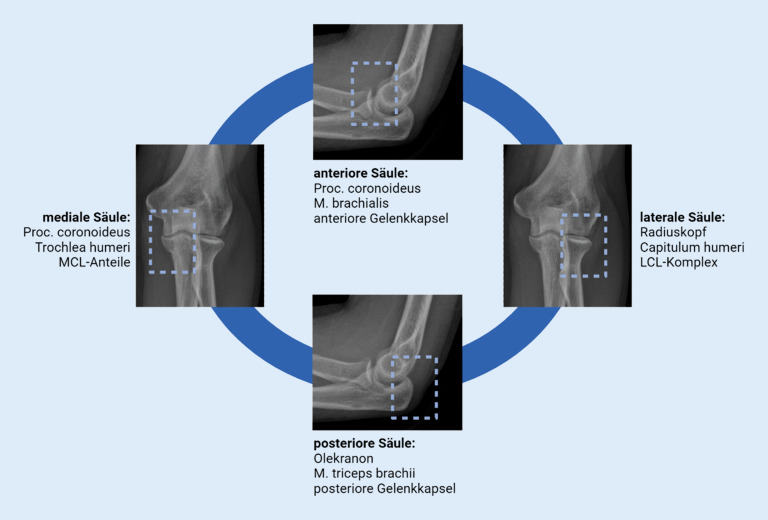

Um evtl. Begleitverletzungen in Beziehung zur Luxationsrichtung zu evaluieren, kann das Stabilitätsmodell nach Ring und Jupiter richtungweisend sein (Abb. 2; [10]). Darin wurden die anteriore Kapsel, das Koronoid und der M. brachialis der anterioren Säule zugeschrieben, wohingegen die dorsale Kapsel, das Olekranon und der M. triceps die posteriore Säule bilden. Lateralseitig bilden das LCL, der Radiuskopf und das Capitulum humeri die radiale Säule, und medial besteht die ulnare Säule aus dem MUCL, dem Koronoid und der Trochlea [10].

### Merke

Das Wissen über die relevanten anatomischen Strukturen am Ellenbogengelenk ist für die optimale Versorgung essenziell.

## Pathomechanismus und Begleitverletzungen

Einfache Ellenbogenluxationen werden zumeist verursacht durch einen Sturz auf die ausgestreckte Hand, der zu einer außenrotierten, valgisierten und axial gerichteten Belastung des Ellenbogens führt. Die Einteilung der **Luxationsrichtung**

**Figure 3:**
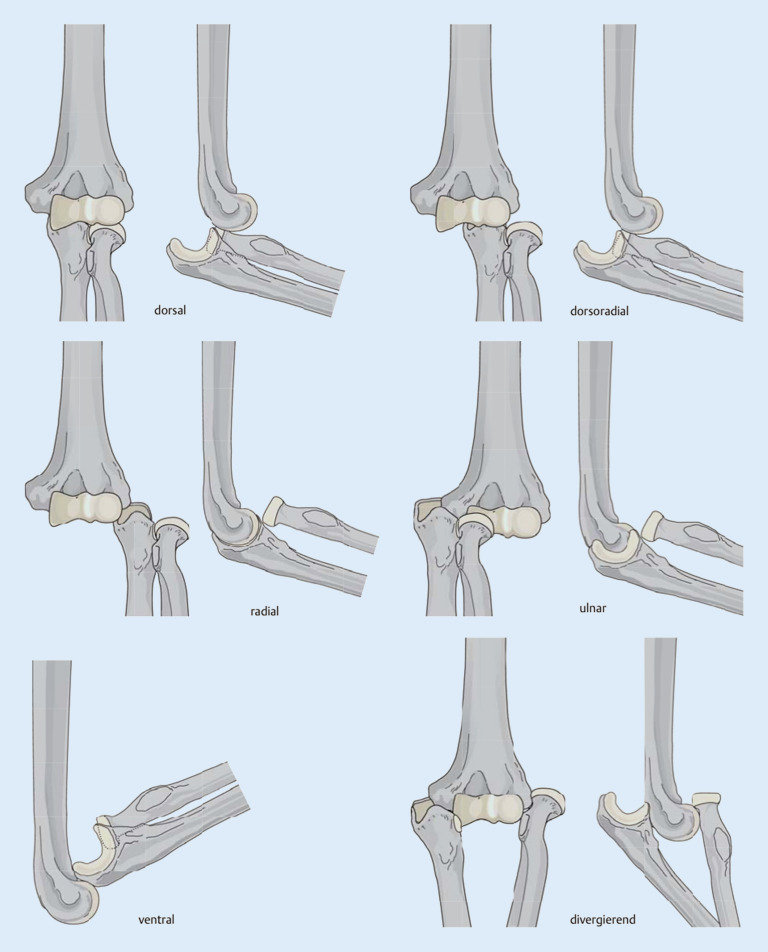

**Figure 4:**
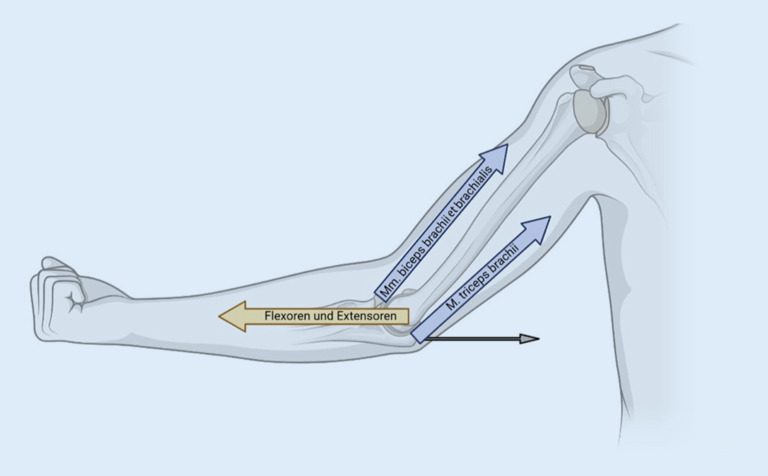

Luxationsrichtung

nach Burkhart et al. [5] ist in Abb. 3 dargestellt. Die häufigsten Luxationen sind mit ca. 80 % nach posterior oder posterolateral gerichtet [12]. Die genannte Richtung beschreibt die Stellung vom Unter- zum Oberarm. Die Mehrheit der posterolateralen Luxationen ist aufgrund der einwirkenden **muskulären Zugkräfte**

muskulären Zugkräfte

an Unter- und Oberarm erklärbar (Abb. 4).

Außerordentlich relevant ist das Vorkommen von Begleitverletzungen. O’Driscoll et al. beschrieben als einen möglichen Erklärungsansatz, dass das typische Verletzungsmuster eine Verletzung des Kapsel-Band-Apparats von lateral nach medial beinhaltet, auch bekannt als **„Horii circle“**

**Figure 5:**
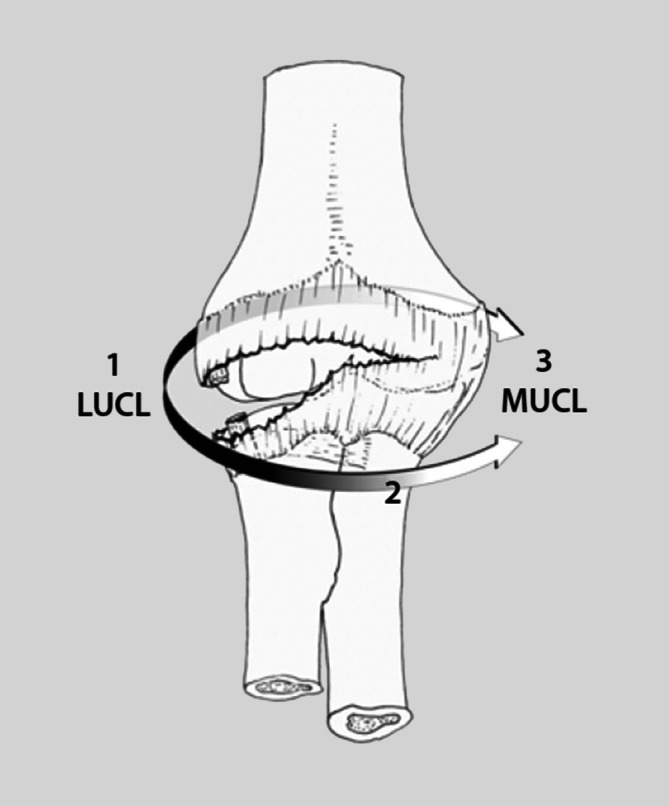

„Horii circle“

(Abb. 5; [9]). Zunächst wird der laterale Kollateralbandkomplex (LCL) von seinem Ursprung am lateralen Epicondylus des Humerus abgelöst, was zu einer posterolateralen Instabilität des Ellenbogens führt. Es folgen Rupturen der vorderen und hinteren Kapsel. Das anteriore Bündel des MUCL wird daraufhin verletzt, was mit einer medialen Instabilität des Ellenbogens einhergeht [9]. Dieser Theorie widersprechen die Ergebnisse einiger Studien, die nach erfolgter Luxation bei den untersuchten Patienten lediglich eine mediale Instabilität beobachten konnten [14, 15].

**Figure 6:**
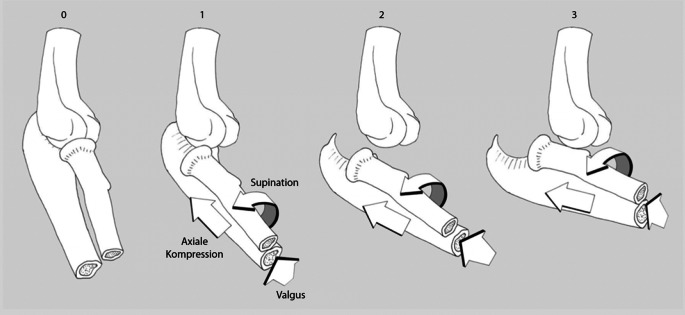

Die Stadieneinteilung der Ellenbogenluxation nach O’Driscoll (Abb. 6) soll die **kapsuloligamentären Begleitverletzungen**

kapsuloligamentären Begleitverletzungen

einbeziehen. Während im reponierten Zustand eine kongruente Gelenkstellung vorhanden ist, beschreibt Stadium 1 eine akute posterolaterale Instabilität bei LUCL-Ruptur. Mit der inkompletten Luxation im Stadium 2 sind eine LUCL-Ruptur sowie Verletzung der dorsalen und ventralen Kapselanteile assoziiert. Als Stadium 3 wird die vollständige Luxation bezeichnet [5, 9].

Rupturen der Kollateralbänder können interligamentär, distal oder proximal auftreten [16]. Neben ligamentären Verletzungen können aufgrund des Luxationsmechanismus oder während der Reposition **osteochondrale Läsionen**

osteochondrale Läsionen

auftreten. Diese sind am häufigsten am dorsalen Capitulum humeri (bei osteochondraler Fraktur als **Osborne-Cotterill-Läsion**

Osborne-Cotterill-Läsion

bezeichnet), am ventralen Radiuskopf, an der Olekranon- oder Koronoidspitze oder an der Trochlea zu finden [16].

Im Folgenden werden ausgewählte relevante, mit Ellenbogenluxationen assoziierte Frakturen, beschrieben. Zur weiteren Vertiefung in das Thema Ellenbogenluxationsfrakturen wird die Lektüre des gleichnamigen Kapitels, verfasst von Hollinger und Lenich, in dem Buch *Expertise Ellenbogen *von Müller et al. [16] empfohlen.

**Koronoidfrakturen**

Koronoidfrakturen

sind relativ seltene Verletzungen, die bei bis zu 15 % der Ellenbogenluxationen auftreten. Regan und Morrey unterteilen in folgende 3 Arten von Koronoidfrakturen: Bei Typ-I-Frakturen ist die Koronoidspitze betroffen, bei Typ-II-Frakturen mehr als nur die Spitze und weniger als 50 % des Koronoids sowie bei Typ-III-Frakturen mehr als 50 % des Koronoids [17, 18, 19]. Für jeden Typ gibt es die Subgruppierung in A und B, wobei das B auf eine damit verbundene Dislokation hinweist. Die stabilisierende Funktion des Koronoids für das Ellenbogengelenk konnte mehrfach bewiesen werden; dieses Wissen sollte bei Koinzidenz mit einer Gelenkluxation eine regelmäßige Evaluation der Gelenkstabilität und Anpassung des therapeutischen Vorgehens nach sich ziehen [20]. Die Kombination einer Radiuskopf- mit einer Koronoidspitzenfraktur weist ein höheres Risiko für persistierende Gelenkinstabilitäten auf als Koronoidfrakturen, die basisnah und groß sind [21].

**Radiuskopffrakturen**

Radiuskopffrakturen

werden nach Mason und modifiziert nach Hotchkiss in folgende 4 Typen eingeteilt und sind eng mit **osteoligamentären Begleitverletzungen**

osteoligamentären Begleitverletzungen

vergesellschaftet [9]. Typ I beschreibt undislozierte Frakturen, Typ II Frakturen mit > 2 mm Dislokation, Typ III mehrfragmentäre Radiuskopffrakturen und Typ IV eine begleitende Luxation des Radiuskopfes. Hierbei ist zu erwähnen, dass bei einer Verletzung des Ellenbogens die Untersuchung des ipsilateralen Handgelenks obligat ist, um weitere Traumafolgen auszuschließen. Eine seltene, aber komplikationsreiche Verletzung ist die **Essex-Lopresti-Läsion**

Essex-Lopresti-Läsion,

die eine Radiuskopffraktur mit Ruptur der Membrana interossea des Unterarms sowie eine Luxation des distalen Radioulnargelenks umfasst [5].

Tritt eine Koronoidfraktur zusammen mit einer posterolateralen Ellenbogenluxation und einer Radiuskopffraktur auf, wird dies als **„Terrible Triad“**

„Terrible Triad“

bezeichnet. Diese Kombination von Verletzungen geht mit einem hohen Risiko für rezidivierende Luxationen, chronische Instabilität und einer **posttraumatischen Arthrose**

posttraumatischen Arthrose

einher [11]. Die Stabilitätskomponente der radialen Säule ist maßgeblich relevant; dies kann bei fälschlich indizierter Radiuskopfresektion folgenschwere Komplikationen nach sich ziehen [16].

### Cave

Die Essex-Lopresti-Läsion und die Terrible Triad am Ellenbogen können leicht übersehen oder in ihren gravierenden Komplikationsfolgen unterschätzt werden.

## Diagnostik

In der akuten Notfallsituation präsentiert sich der Patient nach erfolgtem Trauma typischerweise mit einem schmerzenden Ellenbogengelenk. Als Hinweis kann die klinische **Deformität**

Deformität

oder eine deutliche **Gelenkschwellung**

Gelenkschwellung

dienen; in einigen Fällen kann jedoch hauptsächlich die Anamnese einer bereits reponierten Luxation richtungweisend sein. Nach Beurteilung des neurovaskulären Status und Evaluierung möglicher Begleitverletzungen sollte bei Verdacht auf eine Ellenbogenluxation zeitnah eine **radiologische Bildgebung**

radiologische Bildgebung

mithilfe des seitlichen und anterior-posterioren Röntgenbilds erfolgen [5]. Nach radiographischer Bestätigung des Verdachts einer Luxation sollte schnellstmöglich die Reposition in Analgosedierung durchgeführt werden. Hieran muss sich obligat die klinische Testung der Stabilität anschließen. Als wegweisend für die weitere Therapie ist die **Stabilitätsuntersuchung**

Stabilitätsuntersuchung

in endgradiger Extension und Flexion, wobei der Prüfung einer Luxationstendenz im **funktionellen Bogen**

funktionellen Bogen

(Extension/Flexion 0/30/130°) eine besondere Relevanz zukommt [16]. Zusätzliche Hinweise auf Verletzungen des Bandapparats können **Druckschmerzen**

Druckschmerzen

über den Ansatz- und Insertionspunkten sein sowie Schwellung oder **Hämatomverfärbungen**

Hämatomverfärbungen

im Verlauf der Kollateralbänder [7]. Nach erneuter Erhebung des neurovaskulären Status und Ruhigstellung in einer Oberarmschiene in 90° Stellung muss folglich im **Post-repositionem-Röntgenbild**

**Figure 7:**
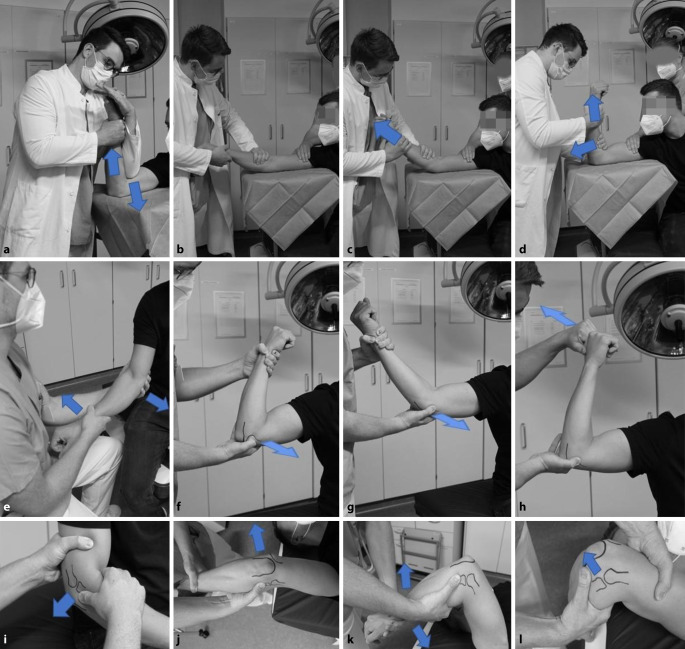

**Figure 8:**
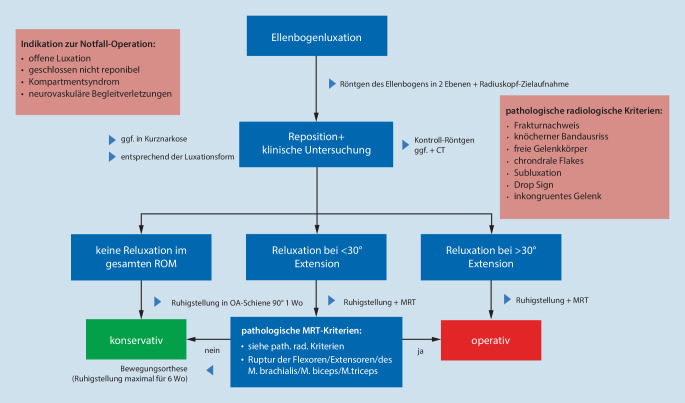

Post-repositionem-Röntgenbild

nach Hinweisen auf Begleitverletzungen gesucht werden. Bei knöchernen Begleitverletzungen oder Instabilitätszeichen (Abb. 8) sollte direkt eine CT-Diagnostik angeschlossen werden, um akut interventionsbedürftige Traumafolgen auszuschließen [16]. Eine **sonographische Beurteilung**

sonographische Beurteilung

des Ellenbogengelenks durch einen erfahrenen Untersucher kann eine **ligamentäre Instabilität**

ligamentäre Instabilität

aufzeigen [22]. Eine klinische und radiologische Reevaluation sollte nach 5 bis 7 Tagen erfolgen, um auf diese Weise eine mögliche Instabilität früh zu detektieren. Neben Testung der Varus- und Valgusstabilität (Abb. 7e) in Extension und aufsteigender Flexion (30° und 60°) unter Bildwandlerkontrolle sollte die posterolaterale Instabilität mithilfe des Pinzettengriffs, Pivot-Shift-Tests und Posterolateral Rotatory Drawer Test (Abb. 7i–l) untersucht werden [23]. Der mediale Bandapparat kann z. B. mithilfe des Moving Valgus Stress Test oder Milking-Manövers überprüft werden (Abb. 7f–h; [23]). Bei bestehendem Verdacht können folglich in einer **magnetresonanztomographischen Untersuchung**

magnetresonanztomographischen Untersuchung

ligamentäre, muskuläre, kapsuläre oder osteochondrale Läsionen diagnostiziert werden [5].

### Merke

Als wegweisender diagnostischer Parameter in der Akutbehandlung der Ellenbogenluxation dient die Stabilitätsprüfung im funktionellen Bogen.

## Therapie

Für das therapeutische Vorgehen empfiehlt sich ein Algorithmus, um das Risiko evtl. Komplikationen oder Folgeschäden zu minimieren (Abb. 8). Dieser sollte jedoch lediglich als Orientierung dienen, da im Einzelfall adaptiert an den Anspruch des Patienten in Bezug auf die körperliche Belastbarkeit unbedingt eine **individualisierte Therapieentscheidung**

individualisierte Therapieentscheidung

getroffen werden muss. Nach der orientierenden Anamneseerhebung, der fokussierten klinischen Untersuchung mit obligater Testung des peripheren neurovaskulären Status sollte schnellstmöglich die Röntgenuntersuchung folgen. Die Richtung der Ellenbogenluxation ist entscheidend für die richtige Reposition. Bei einer Luxation ohne Frakturbeteiligung ist das Hauptziel die sofortige **geschlossene Reposition**

geschlossene Reposition

zur Erlangung der vollständigen Gelenkkongruenz, sodass eine **frühfunktionelle Beübung**

frühfunktionelle Beübung

möglich ist. Im Allgemeinen lässt sich die einfache Ellenbogengelenkluxation gut in einer Kurznarkose reponieren. In Abb. 7a–d sind zwei etablierte und im eigenen Vorgehen favorisierte Techniken zur Reposition einer posterolateralen Luxation dargestellt. Anschließend eignet sich v. a. die Stabilitätstestung in Narkose zur klinischen Untersuchung nach der Reposition (Abb. 7e–l sowie die zuvor beschriebene Stabilitätstestung unter Durchleuchtung). Ereignet sich bei der Stabilitätsprüfung keine Reluxation im endgradigen vollen Bewegungsausmaß, kann bei fehlenden pathologischen radiologischen Kriterien und unauffälligen klinisch-radiologischen Verlaufskontrollen eine **konservative Therapie**

konservative Therapie

gebahnt werden (Abb. 8). Im Fall von Luxationen zwischen endgradiger Extension und 30°-Flexion sind eine engmaschige klinische Reevaluation und die Durchführung einer MRT richtungweisend. Einige Autoren tendieren mittlerweile auch hier zur frühzeitigen operativen Stabilisierung [5]. Bei Reluxation im funktionellen Bogen, einer persistierenden Subluxationsstellung oder intraartikulären pathologischen Veränderungen sollte in jedem Fall eine zeitnahe **operative Versorgung**

operative Versorgung

durchgeführt werden [16].

Die meisten Ellenbogengelenkluxationen können mit sehr guten funktionellen Langzeitergebnissen konservativ therapiert werden [3]. Empfehlenswert ist die Ruhigstellung in 90°-Flexion für eine Woche bis zur Nachkontrolle mit radiologischer Untersuchung und erneuter Stabilitätstestung. Zeigt das Gelenk weiterhin über das gesamte Bewegungsausmaß keine Reluxationstendenz, kann eine frühfunktionelle Beübung erfolgen und eine **Bewegungsorthese**

Bewegungsorthese

für weitere 5 Wochen angelegt werden. Iordens et al. verglichen die frühe Mobilisierung in einer multizentrischen, randomisierten Studie mit der Ruhigstellung im Gips und zeigten, dass Patienten mit früher Beübung nach kürzerer Zeit ein besseres Bewegungsausmaß aufwiesen, ohne dass vermehrte Komplikationen zu verzeichnen waren [6]. Optimierte Nachbehandlungsschemata mit Fokus auf eine frühfunktionelle Beübung für Ellenbogengelenkluxationen erzielen sehr gute funktionelle Ergebnisse [24]. Ein Protokoll für **Überkopfübungen**

Überkopfübungen,

das von Schreiber et al. erstellt wurde, kann eine Woche nach der Verletzung begonnen werden und bewirkt die Umwandlung der Schwerkraft als belastende Kraft in eine stabilisierende Komponente [25].

Bedarf es einer operativen Therapie (Abb. 8), hat sich in den letzten Jahren die **Arthroskopie**

Arthroskopie

des Ellenbogens zur diagnostischen und zur therapeutischen Versorgung weitestgehend etabliert (häufig in Kombination mit offenen operativen Verfahren). Hierdurch können im Akutfall oder bei chronischen pathologischen Veränderungen Hämatome gespült, Arthrolysen durchgeführt, Briden gelöst, Bandinstabilitäten beurteilt (Wechselstabtestung) sowie intraartikuläre Gelenkkörper und Abscherfragmente adressiert werden [26].

Je nach zu adressierender pathologischer Störung muss ein lateraler, medialer oder bilateraler Zugang durchgeführt werden. Lässt die strukturelle Beschaffenheit der rupturierten Bänder eine **primäre Rekonstruktion**

primäre Rekonstruktion

zu, kann das laterale oder mediale Seitenband mithilfe eines Fadenankers refixiert werden. Dies sollte innerhalb von 14 Tagen nach dem Trauma und gemeinsam mit der Refixation der Extensoren- und/oder Flexorenmuskulatur erfolgen [16]. Als weitere Möglichkeit hat sich in den letzten Jahren die Versorgung mithilfe des **„ligament bracing“**

„ligament bracing“

etabliert. Greiner et al. zeigten, dass die Augmentation mit einem nichtresorbierbaren Tape, das nach einer Ellenbogenluxation als „internal bracing“ fungiert, eine direkte postoperative Mobilisierung und Wiederherstellung der Stabilität unter vollem Bewegungsumfang des Ellenbogens ermöglichen kann [27].

Der **Bewegungsfixateur**

Bewegungsfixateur

eignet sich in ausgewählten Fällen mit schwierigen Weichteilverhältnissen, komplexen Luxationsfrakturen, persistierender Instabilität trotz erfolgter Bandnaht oder chronischer Subluxationsstellung. Nach erfolgter Therapie mithilfe des Bewegungsfixateur sind sich gute funktionelle Ergebnisse und niedrige Komplikationsraten zu verzeichnen [28]. Die exakte Anbringung des Fixateur ist jedoch unabdingbar und Grundlage der erfolgreichen Therapie.

Die chronische Ellenbogeninstabilität äußert sich häufig als unspezifischer Ellenbogenschmerz und präsentiert sich in Form einer oft missinterpretierten Epikondylitis-ähnlichen Beschwerdesymptomatik. Bisherige Studien belegen sehr gute Langzeitergebnisse für konservativ therapierte Patienten mit Ellenbogengelenkluxationen trotz persistierender medialer Instabilität [15]. Jedoch fand sich eine Korrelation dieser medialen Instabilität mit zunehmender **degenerativer Gelenkveränderung**

degenerativer Gelenkveränderung

[15]. Deshalb bedarf es einer ausführlichen diagnostischen Abklärung und ggf. arthroskopischen Evaluation, um die richtige Therapie initiieren zu können und das Risiko von Langzeitfolgen gering zu halten. Zur Stabilisierung mithilfe der **Augmentation**

Augmentation

eignet sich der Einsatz von Trizeps-Streifen sowie Grazilis- oder Palmaris-longus-Sehnen als Bandplastik, ggf. unter Applikation eines Bewegungsfixateurs [16].

### Merke

Im Einzelfall muss unbedingt eine individualisierte Therapieentscheidung, die an den Anspruch des Patienten adaptiert ist, getroffen werden.

## Komplikationen

Anders als bei Luxationen der Schulter treten **Rezidivluxationen**

Rezidivluxationen

am Ellenbogen sowohl nach konservativer als auch nach operativer Therapie mit 1–2 % der Fälle nur selten auf [29]. Komplikationsreiche Verläufe sind jedoch auch nach „einfachen“ ligamentären Ellenbogenluxationen keine Seltenheit. Neben neurovaskulären Verletzungen (6 %) und erhöhtem Risiko für eine posttraumatische Kubitalarthrose (7 %) zählen insbesondere **heterotope Ossifikationen**

heterotope Ossifikationen

(HO) und die **posttraumatische Ellenbogensteife**

posttraumatische Ellenbogensteife

zu den typischen Komplikationen [24]. Die Gelenkinstabilität des Ellenbogens wurde bereits im Abschn. „Therapie“ beschrieben.

Da die Stabilität des Ellenbogengelenks in der Nachbehandlung der Ellenbogenluxationen im Fokus steht, wird häufig ein initial restriktives Nachbehandlungsschema angewendet. Jedoch sollte nach 6 bis 12 Wochen der Nachbehandlung die endgradige Flexion und Extension erreicht sein [16]. Ein absoluter Grenzwert für das Vorliegen einer Ellenbogensteife existiert nicht. Morrey et al. stellten bereits 1981 fest, dass die meisten Aktivitäten des täglichen Lebens mit einem Bewegungsausmaß von 100° („funktioneller Bogen“ mit Extension – Flexion 30–130° und Supination – Pronation 50–50°) bewältigt werden können [30]. Eine klinisch relevante Ellenbogensteife tritt bei Ellenbogenluxationen nach Immobilisierung über 3 Wochen in 22 % der Fälle, nach operativer Stabilisierung in 16 % der Fälle und bei frühfunktionell-konservativer Behandlung in 9 % der Fälle auf [24]. Kommt es zu einer posttraumatischen Steife, können Bewegungseinschränkungen (insbesondere Extension und Pro‑/Supination) durch Ausgleichsbewegungen der Schulter und des Rumpfes kompensiert werden. Einschränkungen der Flexion lassen sich jedoch am schwersten ausgleichen. Eine Flexion über 140° ist für Bewegungen der Hand zu Kopf und Gesicht notwendig [31]. Liegt eine deutliche Einschränkung des Bewegungsausmaßes vor, sollten zunächst strukturelle Ursachen der Bewegungseinschränkung (wie **persistierende Subluxationen**

persistierende Subluxationen)

ausgeschlossen werden. Konservative Behandlungen der Ellenbogensteife mithilfe intensiver Physiotherapie und entweder dynamischer oder statischer Schienenbehandlung können bei langer Therapiedauer über 6 bis 12 Monate zu einer Steigerung der Beweglichkeit führen [32]. Im Fall von ausgeprägteren Bewegungseinschränkungen und persistierender Steife nach ausgeschöpfter konservativer Therapie können sowohl offene als auch arthroskopische Verfahren das Bewegungsausmaß deutlich verbessern. In einem systematischen Review von Kodde et al. konnte das Bewegungsausmaß mithilfe offener und arthroskopischer Verfahren um durchschnittlich 51° bzw. 40° gesteigert werden. Die Komplikationsrate war bei arthroskopischen Verfahren mit 5 % im Vergleich zu 23 % bei offenen Verfahren signifikant geringer [33]. Auch anschließend an eine operative Therapie sollte eine intensive frühzeitige Mobilisationsbehandlung erfolgen. In einer randomisierten Untersuchung von O’Driscoll et al. konnten nach arthroskopischer **Arthrolyse**

Arthrolyse

mit einer **Motorschienenbehandlung**

Motorschienenbehandlung

sehr gute Ergebnisse erzielt werden. Die Motorschienengruppe konnte im Vergleich zur konventionellen Physiotherapiegruppe signifikant häufiger ein funktionelles Bewegungsausmaß wiedererlangen [34].

Nach Ellenbogenluxationen können bei ca. 30 % aller Fälle HO beobachtet werden. Die Rate bei Patienten mit immobilisierender Therapie über 2 Wochen (37 %) sowie nach operativer Therapie (49 %) ist signifikant höher als bei den Patienten mit frühfunktioneller Behandlung (20 %; [24]). Zudem sind nicht alle der röntgenologischen Veränderungen klinisch relevant. Dennoch betreffen klinisch einschränkende Ossifikationen jeden 5. Patienten nach einer Ellenbogenluxation [35]. Luxationsfrakturen sind mit einem deutlich höheren Risiko („odds ratio“: 4,87) für HO assoziiert als vergleichbare Frakturen ohne Luxation [35]. Der Luxationsmechanismus sowie die assoziierten Weichteilverletzungen scheinen eine wesentliche Rolle für die Entstehung von HO zu spielen. Ein weiterer unabhängiger Risikofaktor scheint ein verzögerter Operationszeitpunkt zu sein [35]. Die Verteilung der Ossifikationen am Ellenbogen ist mit dem Verletzungsmuster assoziiert, sodass HO nach Luxationen zumeist im Bereich der Kollateralbänder zu finden sind [36].

Zur Prophylaxe der HO wurden die bereits in der Hüftchirurgie bewährten Konzepte auf den Ellenbogen übertragen. So scheinen auch am Ellenbogengelenk die Einnahme von **nichtsteroidalen Antirheumatika**

nichtsteroidalen Antirheumatika

oder alternativ eine **prophylaktische Einzelbestrahlung**

prophylaktische Einzelbestrahlung

mit zumeist 7,0 Gy gleichwertige Ergebnisse zu erzielen [37]. Im Fall einer Luxationsfraktur sollte auf eine Bestrahlung verzichtet werden, da sich in einer randomisierten Studie von Hamid et al. eine signifikant erhöhte Rate an **Pseudarthrosen**

Pseudarthrosen

nach Bestrahlung zeigte; dies führte zum vorzeitigen Abbruch der Studie [38]. Bei Auftreten von klinisch einschränkenden Ossifikationen kann eine deutliche Verbesserung des Bewegungsumfangs durch eine **Exzision**

Exzision

der HO erzielt werden, jedoch ist die Komplikationsrate eines solchen Eingriffs (Nervenläsionen, Wundheilungsstörungen, Rezidivossifikationen) mit ca. 20 % durchaus hoch [39]. Lange wurde propagiert, mit der Exzision bis zur vollständigen Reifung der HO abzuwarten, da dies die Rezidivrate verringern sollte. Neuere Untersuchungen zeigen jedoch, dass auch eine frühzeitige Entfernung der Ossifikationen gleichwertige [40] bis bessere [41] funktionelle Ergebnisse bei gleicher Rezidivrate [40] erzielen kann.

### Cave

Komplikationsreiche Verläufe sind auch nach Ellenbogenluxationen ohne assoziierte Fraktur keine Seltenheit.

## Fazit für die Praxis

- Bei korrekter Behandlung ist die einfache Luxation des Ellenbogens mit guten bis sehr guten Ergebnissen vergesellschaftet.
- Die funktionellen Ergebnisse können maßgeblich durch frühfunktionelle Beübung verbessert werden.
- Eine regelmäßige klinische und radiologische Evaluation nach erfolgter Ellenbogengelenkluxation ist obligat, um mögliche Komplikation zu vermeiden.
- Die klinische Stabilitätstestung zur Detektion jener Ellenbogenverletzungen, die im funktionellen Bogen instabil sind, ist essenziell.
- Die operative Therapie von Ellenbogengelenkluxationen, konsekutiven Instabilitäten und assoziierten Frakturen bedarf eines hohen Maßes an Erfahrung und sollte nach dem Leitsatz „best team, best time“ erfolgen**.**

## CME-Fragebogen

Welche ist die häufigste Luxationsrichtung bei Ellenbogengelenkluxationen?

Ventral

Dorsoradial

Ulnar

Radial

Divergierend

Welche der folgenden operativen Möglichkeiten eignet sich zur chirurgischen Versorgung einer komplexen Bandinstabilität am Ellenbogengelenk?

Zuggurtungsosteosynthese des Epicondylus lateralis

Bewegungsfixateur

Stabilisierungsoperation nach Latarjet

Bandaugmentation der distalen Bizepssehne

Olekranonplattenosteosynthese

Welche dieser Komplikationen kann als eine der häufigeren Folgen nach Ellenbogengelenkluxationen auftreten?

Heterotope Ossifikationen

Instabilität des Akromioklavikulargelenks

Ruptur der Membrana interossea des Unterarms

Radiuskopfnekrose

Karpaltunnelsyndrom

Welche Struktur wird nach O’Driscoll laut Theorie des „Horii circle“ bei einer Luxation im Ellenbogengelenk zuerst verletzt?

Die ventrale Kapsel

Die dorsale Kapsel

Das mediale Kollateralband

Das laterale ulnare Kollateralband

Die Trizepssehne

Welche Verletzung ist per definitionem Teil der Essex-Lopresti-Läsion?

Transolekranonfraktur

Distale Humerusfraktur

Skaphoidfraktur

Radiuskopffraktur

Distale Radiusfraktur

An welcher knöchernen Struktur setzt das anteriore Bündel des „medial ulnar collateral ligament“ (MUCL) an?

Tuberositas radii

Crista supinatoria der Ulna

Tuberculum subliminus der Ulna

Radiuskopf

Capitulum radii

Welche der folgenden Strukturen zählt nach O’Driscoll zu den primären Stabilisatoren am Ellenbogengelenk?

Extensorenmuskulatur

Radiuskopf

Humeroulnargelenk

Anteriore Kapsel

Flexorenmuskulatur

Welches der folgenden Anteile gehört zum lateralen Kollateralbandkomplexes (LCL)?

Das anteriore Bündel (anteromediale Kollateralband)

Das Lig. anulare

Das posteriore Bündel (posteromediale Kollateralband)

Das transversale Bündel

Die Membrana interossea antebrachii

Ein 31-jähriger Patient kommt nach einem Sturz auf den Arm und folglich aufgetretenen starken Schmerzen im linken Ellenbogengelenk sowie einer klinischen Luxationsstellung zu Ihnen in die zentrale Notaufnahme. Was wäre der nächste sinnvolle Schritt?

Als diagnostisches Mittel der Wahl sollte zunächst eine MRT des Ellenbogengelenks durchgeführt werden.

Auch bei isolierter Schmerzsymptomatik am Ellenbogengelenk beinhaltet die klinische Untersuchung den Ausschluss von Begleitverletzungen.

Die Reposition des Ellenbogengelenks sollte ohne Analgetika erfolgen, um die klinische Untersuchung nach der Reposition nicht zu verfälschen.

Nach sofortiger Reposition empfiehlt sich die Ruhigstellung des Oberarms in Streckstellung.

Eine Ellenbogengelenkluxation sollte notfallmäßig im OP unter sterilen Bedingungen versorgt werden.

In Ihrer Sprechstunde stellt sich eine 22-jährige Patientin, die vor einer Woche eine rechtsseitige Ellenbogengelenkluxation erlitten hatte, vor. Die klinische Untersuchung zur Instabilitätsprüfung im Ellenbogengelenk nach erfolgter Luxation ist neben bildgebenden Maßnahmen maßgeblich relevant für die weitere Therapie. Welcher der genannten Tests bietet sich an?

Wenn bis zur endgradigen Extension und Flexion keine Reluxation im Ellenbogengelenk auftritt, kann von einer Unversehrtheit des Lig. anulare ausgegangen werden.

Bei jungen, sportlichen Patienten mit hohem körperlichen Anspruch besitzt die Reluxation im funktionellen Bogen des Ellenbogengelenks wenig Aussagekraft.

Der Valgusstresstest überprüft vorwiegend die Instabilität des lateralen Kollateralbands bei gestrecktem Ellenbogengelenk.

Der Pivot-Shift-Test wird in Rückenlage des Patienten mit dem Arm in Überkopfhaltung durchgeführt und prüft eine posterolaterale Instabilität am Ellenbogengelenk.

Mithilfe des Pinzettengriffs, bei dem der Daumen den Zeigefinger berühren muss, wird die Funktionalität des N. ulnaris nach einer Ellenbogengelenkluxation überprüft.

## References

[1] Josefsson PO, Nilsson BE (1986). Incidence of elbow dislocation. Acta Orthop Scand.

[2] de Haan J, Schep NW, Zengerink I, van Buijtenen J, Tuinebreijer WE, den Hartog D (2010). Dislocation of the elbow: a retrospective multicentre study of 86 patients. Open Orthop J.

[3] De Haan J, Schep NW, Tuinebreijer WE, Patka P, den Hartog D (2010). Simple elbow dislocations: a systematic review of the literature. Arch Orthop Trauma Surg.

[4] Royle SG (1991). Posterior dislocation of the elbow. Clin Orthop Relat Res.

[5] Burkhart KJ, Hollinger B, Wegmann K, Müller LP (2012). Luxationen und Bandverletzungen am Ellenbogen und Unterarm. Orthop Unfall Up2date.

[6] Iordens GIT, Van Lieshout EMM, Schep NWL (2017). Early mobilisation versus plaster immobilisation of simple elbow dislocations: results of the FuncSiE multicentre randomised clinical trial. Br J Sports Med.

[7] Mittlmeier T, Beck M (2009). Luxation des Ellenbogengelenks des Erwachsenen. Unfallchirurg.

[8] Ball CM, Galatz LM, Yamaguchi K (2002). Elbow instability: treatment strategies and emerging concepts. Instr Course Lect.

[9] O’Driscoll SW, Jupiter JB, King GJ, Hotchkiss RN, Morrey BF (2001). The unstable elbow. Instr Course Lect.

[10] Ring D, Jupiter JB (1998). Current concepts review - fracture-dislocation of the elbow. J Bone Joint Surg Am.

[11] Ring D, Jupiter JB, Zilberfarb J (2002). Posterior dislocation of the elbow with fractures of the radial head and coronoid. J Bone Joint Surg Am.

[12] Mühlenfeld N, Frank J, Lustenberger T, Marzi I, Sander AL (2022). Epidemiology and treatment of acute elbow dislocations: current concept based on primary surgical ligament repair of unstable simple elbow dislocations. Eur J Trauma Emerg Surg.

[13] Letsch R, Schmitt-Neuerburg KP, Schmit-Neuerburg K-P, Towfigh H, Letsch R (2001). Bandverletzungen und Luxationen des Ellenbogengelenkes. Tscherne Unfallchirurgie: Ellenbogen, Unterarm, Hand.

[14] Kerschbaum M, Thiele K, Scheibel M (2017). Residual increased valgus stress angulation and posterolateral rotatory translation after simple elbow dislocation. Knee Surg Sports Traumatol Arthrosc.

[15] Eygendaal D, Verdegaal SH, Obermann WR, van Vugt AB, Pöll RG, Rozing PM (2000). Posterolateral dislocation of the elbow joint. Relationship to medial instability. J Bone Joint Surg Am.

[16] Müller LP, Hollinger B, Burkhart KJ (2016). Expertise Ellenbogen.

[17] Regan W, Morrey B (1989). Fractures of the coronoid process of the ulna. J Bone Joint Surg Am.

[18] Morrey BF (2000). The elbow and its disorders.

[19] Wells J, Ablove RH (2008). Coronoid fractures of the elbow. Clin Med Res.

[20] Bellato E, Kim Y, Fitzsimmons JS, Berglund LJ, Hooke AW, Bachman DR, O’Driscoll SW (2017). Coronoid reconstruction using osteochondral grafts: a biomechanical study. J Shoulder Elbow Surg.

[21] Terada N, Yamada H, Seki T, Urabe T, Takayama S (2000). The importance of reducing small fractures of the coronoid process in the treatment of unstable elbow dislocation. J Shoulder Elbow Surg.

[22] Kirschbaum S, Plachel F, Kerschbaum M, Gerhard C, Thiele K (2021). Does sonography allow an objective and reproducible distinction between stable, hypermobile, and unstable elbow joints?. J Shoulder Elbow Surg.

[23] Geyer S, Lenich A, Siebenlist S, Engelhardt M, Raschke MJ (2020). Ellenbogenluxationen. Orthopädie und Unfallchirurgie.

[24] Schubert I, Strohm PC, Maier D, Zwingmann J (2021). Simple traumatic elbow dislocations; benefit from early functional rehabilitation: A systematic review with meta-analysis including PRISMA criteria. Medicine (Baltimore).

[25] Schreiber JJ, Paul S, Hotchkiss RN (2015). Conservative management of elbow dislocations with an overhead motion protocol. J Hand Surg Am.

[26] Batko BD, Hakakian D, Norin JL, Tauro JC (2022). Complications in elbow arthroscopy: Management and prevention. Sports Med Arthrosc.

[27] Greiner S, Koch M, Kerschbaum M, Bhide PP (2019). Repair and augmentation of the lateral collateral ligament complex using internal bracing in dislocations and fracture dislocations of the elbow restores stability and allows early rehabilitation. Knee Surg Sports Traumatol Arthrosc.

[28] Meccariello L, Caiaffa V, Mader K, Prkic A, Eygendaal D, Bisaccia M, Pica G, Utrilla-Hernando S, Pica R, Rollo G (2022). Treatment of unstable elbow injuries with a hinged elbow fixator: Subjective and objective results. Strategies Trauma Limb Reconstr.

[29] Catapano M, Pupic N, Multani I, Wasserstein D, Henry P (2022). Early functional mobilization for non-operative treatment of simple elbow dislocations: a systematic review. Shoulder Elbow.

[30] Morrey BF, Askew LJ, Chao EY (1981). A biomechanical study of normal functional elbow motion. J Bone Joint Surg Am.

[31] Raiss P, Rettig O, Wolf S, Loew M, Kasten P (2007). Das Bewegungsausmass der Schulter und des Ellenbogens bei Alltagsbewegungen in der 3D-Bewegungsanalyse. Z Orthop Unfall.

[32] Lindenhovius AL, Doornberg JN, Brouwer KM, Jupiter JB, Mudgal CS, Ring D (2012). A prospective randomized controlled trial of dynamic versus static progressive elbow splinting for posttraumatic elbow stiffness. J Bone Joint Surg Am.

[33] Kodde IF, van Rijn J, van den Bekerom MP, Eygendaal D (2013). Surgical treatment of post-traumatic elbow stiffness: a systematic review. J Shoulder Elbow Surg.

[34] O’Driscoll SW, Lievano JR, Morrey ME, Sanchez-Sotelo J, Shukla DR, Olson TS, Fitzsimmons JS, Vaichinger AM, Shields MN (2022). Prospective randomized trial of continuous passive motion versus physical therapy after arthroscopic release of elbow contracture. J Bone Joint Surg Am.

[35] Hong CC, Nashi N, Hey HW, Chee YH, Murphy D (2015). Clinically relevant heterotopic ossification after elbow fracture surgery: a risk factors study. Orthop Traumatol Surg Res.

[36] Wahl EP, Casey PM, Risoli T, Green CL, Richard MJ, Ruch DS (2021). Heterotopic ossification formation after fractures about the elbow. Eur J Orthop Surg Traumatol.

[37] Henstenburg JM, Sherman M, Ilyas AM (2021). Comparing options for heterotopic ossification prophylaxis following elbow trauma: A systematic review and meta-analysis. J Hand Microsurg.

[38] Hamid N, Ashraf N, Bosse MJ, Connor PM, Kellam JF, Sims SH, Stull DE, Jeray KJ, Hymes RA, Lowe TJ (2010). Radiation therapy for heterotopic ossification prophylaxis acutely after elbow trauma: a prospective randomized study. J Bone Joint Surg Am.

[39] Lee EK, Namdari S, Hosalkar HS, Keenan MA, Baldwin KD (2012). Clinical results of the excision of heterotopic bone around the elbow: a systematic review. J Shoulder Elbow Surg.

[40] Chen S, Yu SY, Yan H, Cai JY, Ouyang Y, Ruan HJ, Fan CY (2015). The time point in surgical excision of heterotopic ossification of post-traumatic stiff elbow: recommendation for early excision followed by early exercise. J Shoulder Elbow Surg.

[41] He SK, Yi M, Zhong G, Cen SQ, Chen JL, Huang FG (2018). Appropriate excision time of heterotopic ossification in elbow caused by trauma. Acta Orthop Traumatol Turc.

